# Health care worker seromonitoring reveals complex relationships between common coronavirus antibodies and COVID-19 symptom duration

**DOI:** 10.1172/jci.insight.150449

**Published:** 2021-08-23

**Authors:** Sigrid Gouma, Madison E. Weirick, Marcus J. Bolton, Claudia P. Arevalo, Eileen C. Goodwin, Elizabeth M. Anderson, Christopher M. McAllister, Shannon R. Christensen, Debora Dunbar, Danielle Fiore, Amanda Brock, JoEllen Weaver, John Millar, Stephanie DerOhannessian, The UPenn COVID Processing Unit, Ian Frank, Daniel J. Rader, E. John Wherry, Scott E. Hensley

**Affiliations:** 1 Department of Microbiology,; 2 Division of Infectious Diseases,; 3 Department of Psychiatry,; 4 Institute for Translational Medicine and Therapeutics, and; 5 Departments of Genetics and Medicine, Perelman School of Medicine, University of Pennsylvania, Philadelphia, Pennsylvania, USA.; 6 The UPenn COVID Processing Unit is detailed in Supplemental Acknowledgments.; 7 Institute for Immunology, Perelman School of Medicine, University of Pennsylvania, Philadelphia, Pennsylvania, USA.

**Keywords:** COVID-19, Immunology, Adaptive immunity

## Abstract

Some studies suggest that recent common coronavirus (CCV) infections are associated with reduced COVID-19 severity upon SARS-CoV-2 infection. We completed serological assays using samples collected from health care workers to identify antibody types associated with SARS-CoV-2 protection and COVID-19 symptom duration. Rare SARS-CoV-2 cross-reactive antibodies elicited by past CCV infections were not associated with protection; however, the duration of symptoms following SARS-CoV-2 infections was significantly reduced in individuals with higher common betacoronavirus (βCoV) antibody titers. Since antibody titers decline over time after CCV infections, individuals in our cohort with higher βCoV antibody titers were more likely recently infected with common βCoVs compared with individuals with lower antibody titers. Therefore, our data suggest that recent βCoV infections potentially limit the duration of symptoms following SARS-CoV-2 infections through mechanisms that do not involve cross-reactive antibodies. Our data are consistent with the emerging hypothesis that cellular immune responses elicited by recent common βCoV infections transiently reduce symptom duration following SARS-CoV-2 infections.

## Graphical abstract

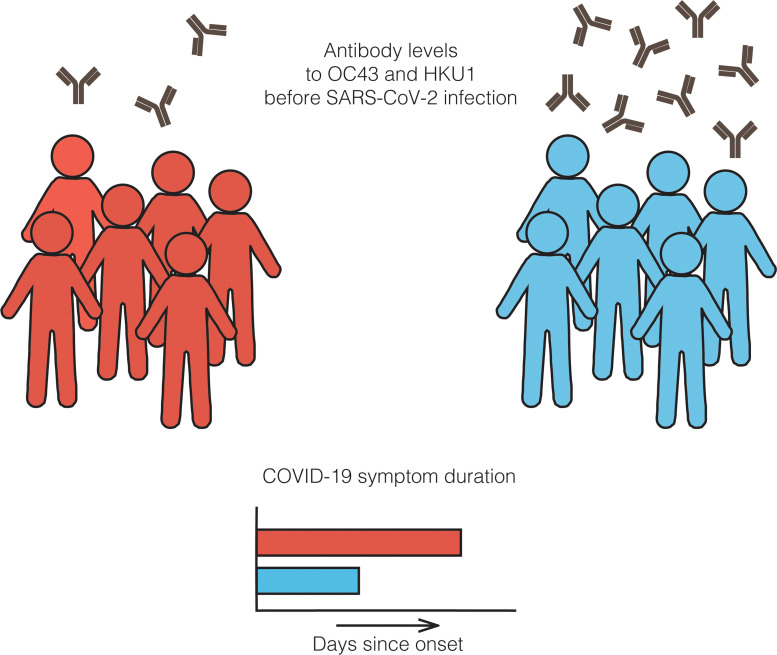

## Introduction

SARS-CoV-2 causes heterogenous disease outcomes in different individuals that can range from asymptomatic infections to critical illness and death (1). It is unknown if prior exposure histories to common coronaviruses (CCVs) contribute to diverse outcomes following SARS-CoV-2 infections. A study reviewing electronic health records indicated that individuals recently infected with CCVs were not protected from SARS-CoV-2 infections but experienced less severe disease upon infection (2). This study found that individuals with recent CCV infections were less likely to require intensive care admission and die following SARS-CoV-2 infections. Our group and others have found that some individuals possessed prepandemic antibodies that cross-react against SARS-CoV-2 (3–5), but these cross-reactive antibodies were not associated with SARS-CoV-2 protection or attenuating COVID-19 severity. Thus, it is unclear how prior CCV exposures influence outcomes following SARS-CoV-2 infections.

Antibody titers against CCVs are elevated after recent CCV infections but then gradually decline over time (6). Antibody titers against CCVs can therefore serve as an “immunological stamp” that dates recent CVV infections. Much less is known about the kinetics of T cell responses following CCV infections and how cellular immunity elicited by past CCV exposures affects subsequent encounters with CCVs and SARS-CoV-2. Some individuals possessed SARS-CoV-2–reactive CD4^+^ and CD8^+^ T cells before the COVID-19 pandemic (7–11); however, the impact of cellular immunity elicited by prior CCV infections on SARS-CoV-2 infections is poorly understood.

In this study, we established a cohort of 2043 health care workers and longitudinally collected serum samples in the spring and summer of 2020 during the first wave of SARS-CoV-2 activity in Philadelphia, Pennsylvania, USA. We identified a subset of health care workers who went on to become infected with SARS-CoV-2 after we collected serum samples. We completed a series of serological assays to determine whether antibodies reactive to SARS-CoV-2 and CCVs were associated with SARS-CoV-2 protection and COVID-19 symptom duration upon infection.

## Results

### Establishment of a health care worker cohort.

**Figure 1:**
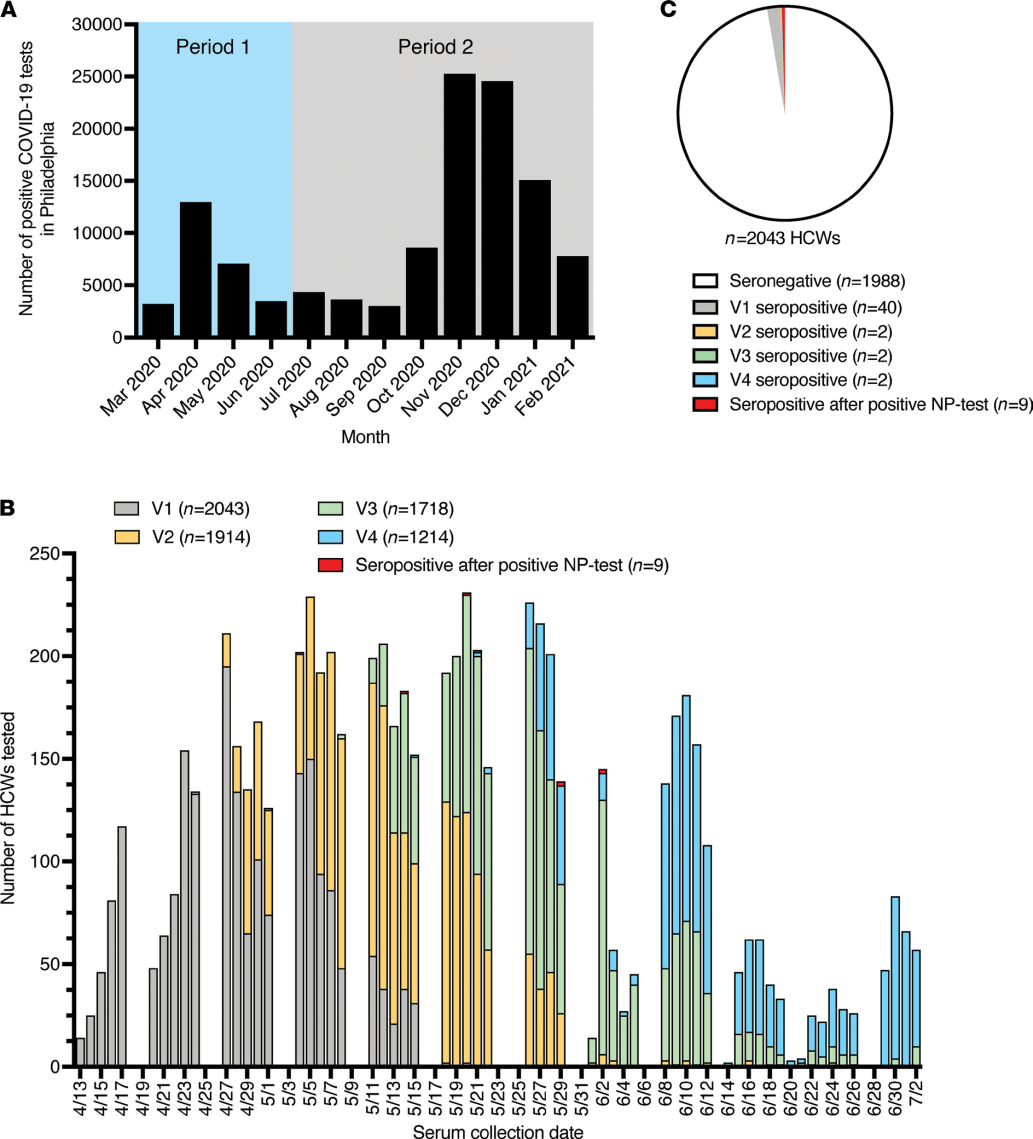
Seropositive health care workers by study visit in relation to SARS-CoV circulation in Philadelphia. (**A**) The number of positive COVID-19 tests in Philadelphia from March 2020 to February 2021 (24). The first viral period was defined as constituting infections that occurred before July 2, 2020, and the second viral period was defined as constituting infections that occurred after July 2, 2020. (**B**) The number of health care workers tested by serum collection date and stratified by study visit and seropositivity status. One of the nine health care workers with a positive NP SARS-CoV-2 PCR test outside of our study seroconverted after July 2, 2020, and their seropositive sample is therefore not shown in this graph. (**C**) Seropositive health care workers (*n* = 55) by study visit. The majority of health care workers (*n* = 1988) were seronegative throughout the study period. HCWs, health care workers; NP, nasopharyngeal; V1, visit 1; V2, visit 2; V3, visit 3; V4, visit 4.

We established a prospective cohort of 2043 health care workers during the spring of 2020 to monitor SARS-CoV-2 seroprevalence and identify correlates of protection against SARS-CoV-2 infections. We included health care workers at 3 hospitals in the University of Pennsylvania Health System (Hospital of the University of Pennsylvania, Penn Presbyterian Medical Center, and Pennsylvania Hospital) who had direct contact with or worked on units with patients, and we excluded anyone previously diagnosed with COVID-19. Participants were predominantly female (75.2%), White (82.9%), and non-Hispanic (96.5%). The median age was 36 years (IQR, 30–46 years) (Supplemental Table 1; supplemental material available online with this article; https://doi.org/10.1172/jci.insight.150449DS1). Participants of our study were enrolled during the spring of 2020 when SARS-CoV-2 began widely circulating in Philadelphia (Figure 1A).

We collected baseline serum samples from each participant between April 13, 2020, and May 20, 2020 (Figure 1B). Within 36–48 hours after sample collection, we quantified levels of SARS-CoV-2 spike receptor-binding domain (S-RBD) serum IgG and IgM antibodies. We collected nasopharyngeal (NP) swabs from all SARS-CoV-2 S-RBD–seropositive participants, and we completed SARS-CoV-2 PCR testing to identify active or recent infections. Participants who were seronegative at the baseline visit were invited for follow-up visits every 2 weeks until July 2, 2020, and NP SARS-CoV-2 PCR testing was completed on all participants who seroconverted. In total, we collected 6897 serum samples between April 13, 2020, and July 2, 2020, from 2043 health care workers (Figure 1B). This included 2043 serum samples collected at a baseline visit, 1914 samples collected at visit 2, 1718 samples collected at visit 3, and 1214 samples collected at visit 4. We also collected serum samples from 8 participants who were seronegative at their baseline visit and had a positive NP SARS-CoV-2 PCR test outside of our study before July 20, 2020. Additional serum samples were collected from seropositive health care workers up to 236 days after seroconversion to monitor the longevity of antibody responses following SARS-CoV-2 infection, including one seropositive participant who had a positive NP SARS-CoV-2 PCR after July 2, 2020.

### Seroprevalence during the first spring/summer 2020 wave of SARS-CoV-2 infections in Philadelphia.

We found that 40 of 2043 health care workers (2.0%) in our cohort possessed serum IgG and/or IgM SARS-CoV-2 S-RBD–reactive antibodies at baseline (Figure 1C). Of the 40 seropositive samples, 17 (42.5%) were IgG^+^/IgM^–^, 7 (17.5%) were IgG^–^/IgM^+^, and 16 (40.0%) were IgG^+^/IgM^+^. Seropositivity remained consistently low throughout the study period (Figure 1B). Of the 2003 health care workers who were seronegative at baseline, 15 health care workers (0.7%) became seropositive on subsequent study visits (Figure 1C). Of the 15 health care workers who seroconverted while enrolled in our study, 5 (33.3%) were IgG^+^/IgM^–^ and 10 (66.7%) were IgG^+^/IgM^+^. As of July 2, 2020, the overall seropositivity rate in our health care workers cohort was 2.7%, which was similar to the 3.2% seroprevalence rate reported for the Philadelphia metro area and surrounding counties from April 13 to April 25, 2020 (12) but lower than the 6.2% seroprevalence rate we previously reported in samples collected from parturient women from April 4, 2020, to June 3, 2020 (13). Consistent with other reports (14, 15), the low seroprevalence within our cohort suggests that PPE and other precautions taken within our hospitals limited the spread of SARS-CoV-2 infections of health care workers.

Of the 40 health care workers who were seropositive at baseline, only 6 (15.0%) had a positive NP SARS-CoV-2 PCR test. As we only enrolled health care workers who did not have a known or suspected history of COVID-19 diagnosis, most seropositive participants entering our study likely had prior asymptomatic SARS-CoV-2 infections before the study began. Of the 15 health care workers who seroconverted after the baseline visit, 12 (80.0%) had a positive NP SARS-CoV-2 PCR test.

### Antibody kinetics after SARS-CoV-2 infection.

**Figure 2:**
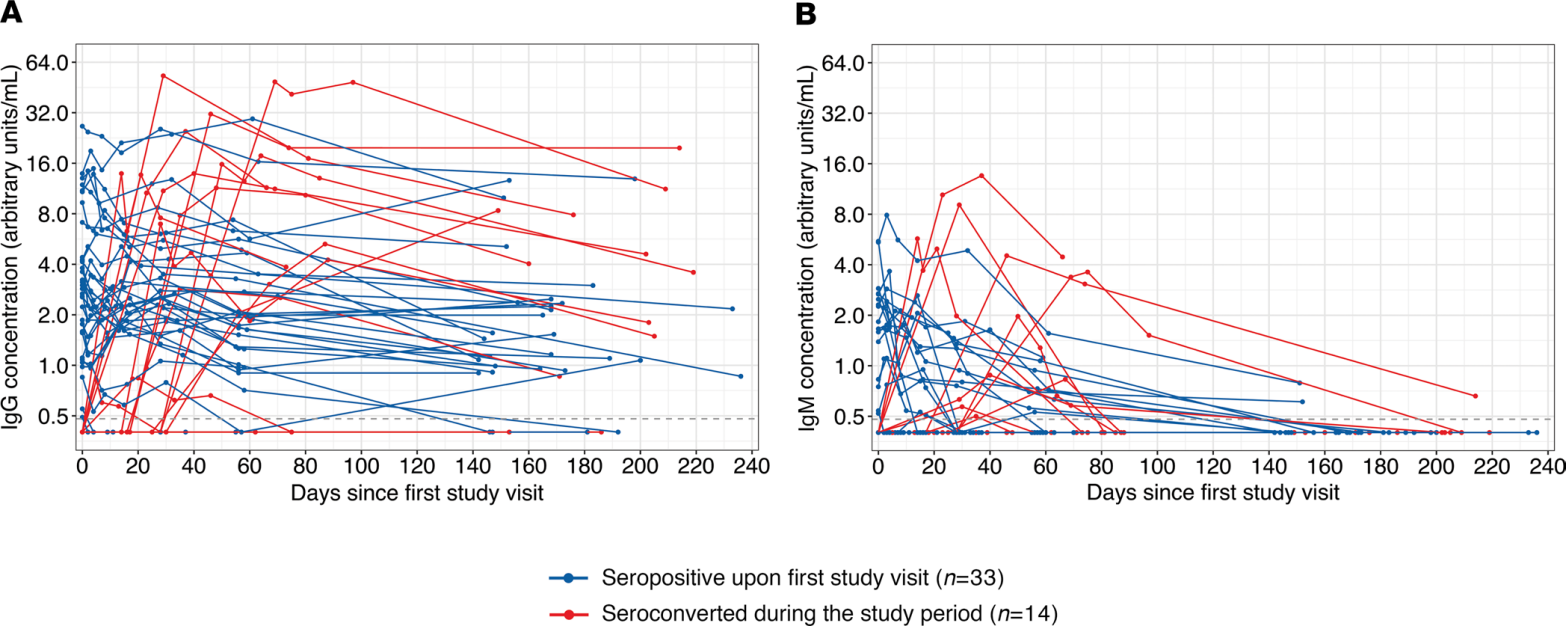
Antibody kinetics in 47 health care workers following SARS-CoV-2 infection (*n* = 300 samples). (**A**) IgG and (**B**) IgM antibodies against the SARS-CoV-2 S-RBD are shown in health care workers who possessed IgG antibodies at the first study visit (*n* = 33) or seroconverted during the study period (*n* = 14). Lines connect samples collected from 1 individual.

We stopped collecting blood samples from seronegative participants in July of 2020 after the first SARS-CoV-2 wave in Philadelphia; however, we continued to collect samples from seropositive health care workers so that we could measure serum antibody levels within infected individuals over time. We quantified SARS-CoV-2 S-RBD antibody levels in samples longitudinally collected from 47 seropositive participants who possessed IgG antibodies, including 33 health care workers who possessed IgG antibodies against SARS-CoV-2 S-RBD at the first study visit and 14 health care workers who seroconverted after first sample collection (Figure 2). Consistent with previous reports (16–19), SARS-CoV-2 S-RBD IgG levels remained relatively stable in the sera of the majority of health care workers (Figure 2A). Of the 39 health care workers with samples collected for 140 days after seroconversion, 33 possessed detectable levels of serum IgG antibodies. Health care workers with undetectable IgG concentrations at 140 days after seroconversion had significantly lower peak geometric mean IgG concentrations compared with health care workers who possessed IgG antibodies at 140 days after seroconversion (0.66 versus 6.35 arbitrary units/mL; *P* < 0.001, unpaired *t* test on log_2_-transformed data). In contrast to IgG levels, the longevity of the IgM antibody response was highly variable among participants (Figure 2B). We detected serum IgM in some participants for multiple weeks, including 1 participant with detectable IgM up to 168 days after seroconversion. Interestingly, 5 of 14 participants who seroconverted did not possess detectable serum IgM at any study visit (Figure 2B).

### Correlates of protection against SARS-CoV-2 infection and disease severity.

SARS-CoV-2 circulated at low levels in Philadelphia during the summer of 2020, but infections increased during the fall of 2020 and the subsequent winter (Figure 1A). We invited all participants to complete an online survey in January 2021 to report SARS-CoV-2 infections since the last blood draw, and over half of the participants (1159 in total) completed this survey. For purposes of this study, we defined infections that occurred during our initial spring and summer sampling period as “viral period 1” (infections that occurred before July 2, 2020) and infections that occurred after our initial sampling period as “viral period 2” (infections that occurred after July 2, 2020) (Figure 1A). We analyzed the last spring and summer serum sample collected from participants who were either infected or not infected during the second viral period to identify specific types of antibodies that were correlated with protection.

Forty-four of the fifty-five health care workers who were SARS-CoV-2 S-RBD seropositive during the first wave of SARS-CoV-2 infections responded to our January 2021 survey. Of 44 participants who had detectable antibodies against the SARS-CoV-2 S-RBD, one seropositive participant (2.3%) reported a PCR-confirmed infection with SARS-CoV-2 during the second viral period in the fall of 2020. This participant experienced COVID-19 symptoms, including cough and difficulty breathing. This participant entered our study as SARS-CoV-2 seropositive and SARS-CoV-2 PCR negative, and therefore, we could not confirm that this participant had a SARS-CoV-2 infection during the first wave of virus circulation in the spring of 2020. It is possible that this individual was not previously infected with SARS-CoV-2 but instead possessed prepandemic cross-reactive S-RBD antibodies, which we have found were present in approximately 0.9% of individuals before the COVID-19 pandemic began (3). It is also possible that this individual was SARS-CoV-2 infected in the spring of 2020 and then reinfected with an antigenically distinct strain of SARS-CoV-2 in the fall of 2020, although we could not investigate this possibility because we were unable to obtain NP samples from the fall infection for sequencing.

**Figure 3:**
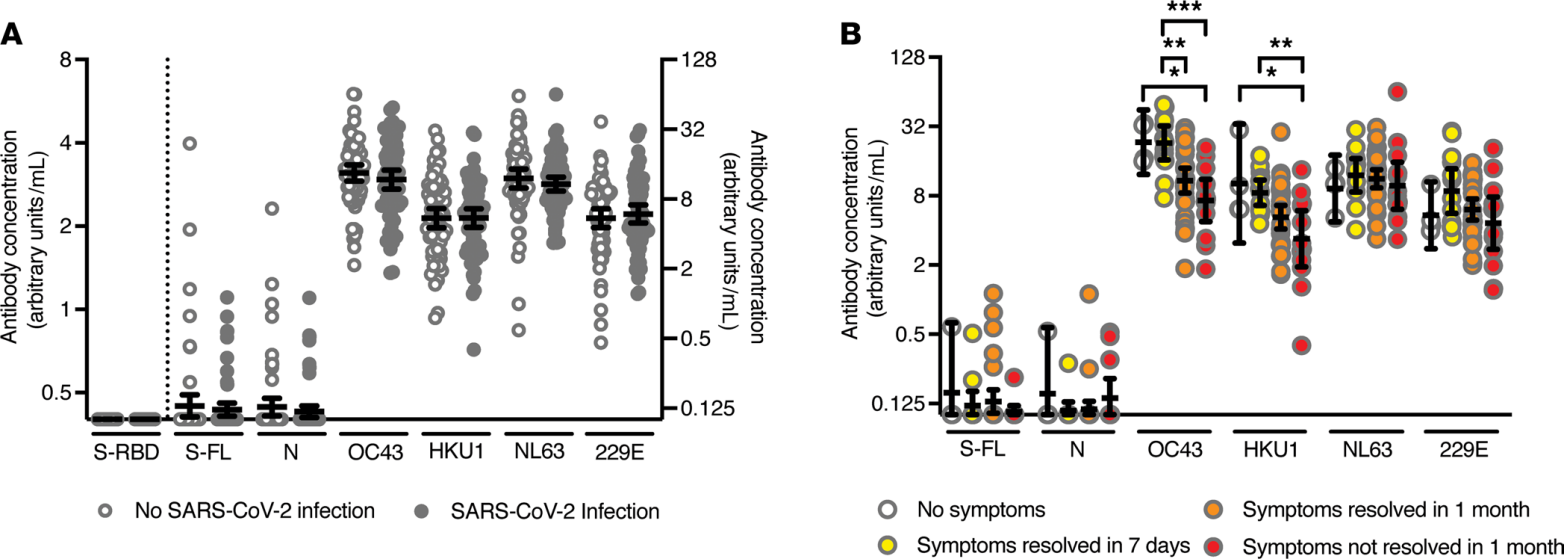
Correlation between preexisting antibody concentrations and reported SARS-CoV-2 infections and duration of COVID-19 symptoms. (**A**) Preexisting antibody concentrations in health care workers with (*n* = 68) and without (*n* = 68) SARS-CoV-2 infection after last blood draw. The control group without SARS-CoV-2 infection after the last blood draw was matched to the infection group based on age and sex. Antibody concentrations were similar between infected and uninfected individuals (*P* > 0.28, unpaired *t* tests using log_2_-transformed antibody concentrations). Antibody concentrations specific to S-RBD are shown on the left *y* axis, and values below the cutoff (0.48 arbitrary units/mL) are set at 0.40 arbitrary units/mL. All other antibody concentrations are shown on the right *y* axis. Values below the limit of detection (0.20 arbitrary units/mL) are set at 0.10 arbitrary units/mL. Horizontal lines show the geometric mean concentrations and 95% confidence intervals. (**B**) Preexisting antibody concentrations in health care workers who reported a PCR-confirmed SARS-CoV-2 infection and had no symptoms (*n* = 4) or indicated symptom duration via an online survey (*n* = 58). Symptom duration are as follows: symptoms resolved within 7 days (*n* = 13), symptoms resolved within 1 month (*n* = 32), and symptoms not resolved within 1 month (*n* = 13). Significant *P* values (*P* < 0.05) are indicated above the graph (*P < 0.05; **P < 0.01; ***P < 0.001, 1-way ANOVA using log_2_-transformed antibody concentrations). Horizontal lines show the geometric mean concentrations and 95% confidence intervals.

Of the 1115 health care workers who did not have detectable SARS-CoV-2 S-RBD antibodies during the spring and summer of 2020 and who responded to our January 2021 online survey, 68 participants (6.1%) reported a lab-confirmed SARS-CoV-2 infection after the last blood draw, including 64 symptomatic infections. Two participants were hospitalized during the fall and winter because of COVID-19 (Supplemental Table 2). We completed additional serological assays using samples collected during the spring and summer of 2020 from the 68 SARS-CoV-2 S-RBD–seronegative individuals who had infections confirmed by PCR during the second viral period and 68 participants matched by age and sex who did not report SARS-CoV-2 infections after the last blood draw. This allowed us to evaluate correlates of protection associated with preventing SARS-CoV-2 infections in individuals who have not been previously exposed to the virus. We used ELISA to quantify IgG antibodies against the SARS-CoV-2 full-length spike (S-FL) protein and N protein as well as antibodies against S-FL proteins from CCVs. Consistent with our previous study (3), we found that preinfection IgG antibodies reactive to the SARS-CoV-2 S-FL protein and N protein were rare and at similar levels in health care workers who were and were not infected with SARS-CoV-2 during the second viral period (Figure 3A). Similarly, we found that IgG antibody titers against S-FL from CCVs were not associated with protection from PCR-confirmed SARS-CoV-2 infections (Figure 3A).

Next, we compared the relationship of preinfection IgG antibody levels with disease severity following SARS-CoV-2 infection using samples collected from SARS-CoV-2 S-RBD seronegative individuals who became infected during the second viral period. Our analysis included samples from 4 health care workers who reported asymptomatic SARS-CoV-2 infections and 58 participants who reported symptomatic SARS-CoV-2 infections (6 participants did not include information about symptoms and were therefore not included in the analyses). We found no correlation between symptom duration and preinfection IgG antibody levels for antibodies reactive to SARS-CoV-2 S-FL and N proteins (Figure 3B). In contrast, we found a strong negative association of symptom duration and preinfection IgG antibody titers against OC43 and HKU1 S-FL proteins (Figure 3B). After adjusting via multivariate regression for age and sex, individuals with higher OC43 and HKU1 S-FL IgG antibody titers had significantly fewer symptomatic days following SARS-CoV-2 infection (*P* = 0.004 and *P* = 0.030, respectively; Supplemental Table 3). There was no correlation between symptom duration and IgG antibody titers against 229E and NL63 S-FL proteins (Figure 3B). This is interesting because 229E and NL63 are both alphacoronaviruses (αCoVs), whereas SARS-CoV-2, OC43, and HKU1 are all betacoronaviruses (βCoVs) (20). While we found significant associations between OC43 and HKU1 IgG antibody titers and COVID-19 symptom duration, we found few associations with preinfection IgG antibody titers and specific symptoms (Supplemental Figure 1). As IgG antibodies against CCVs are elevated after CCV infection and then slowly decline over time (6), individuals with higher OC43 and HKU1 IgG antibody titers in our cohort were more likely recently infected with these common βCoVs. The mechanism underlying the apparent transient cross-protection afforded by recent common βCoV infections is unknown but potentially involves cellular immunity because we found no association with preexisting SARS-CoV-2–reactive antibodies and symptom duration following SARS-CoV-2 infections.

## Discussion

In this study, we show that SARS-CoV-2 infections among health care workers at the University of Pennsylvania are relatively uncommon. Our study, along with those of others (14, 15), suggests that PPE and other precautions have efficiently limited the spread of SARS-CoV-2 within our hospitals. Consistent with the results of other studies (16–19), we show that antibody responses elicited by SARS-CoV-2 are long lived and detectable up to 140 days following infection in the majority of individuals. We identified 1 individual in our study who was potentially infected twice with SARS-CoV-2, but it is unclear whether this individual was truly infected during the first wave of SARS-CoV-2 in Philadelphia. This individual was SARS-CoV-2 S-RBD antibody positive entering the study in the spring of 2020, and it is possible that this participant was not infected during the first SARS-CoV-2 wave but instead possessed prepandemic cross-reactive SARS-CoV-2 S-RBD antibodies that were present in approximately 0.9% of individuals before the pandemic began (3).

The primary goals of our study were initially to use serology to monitor asymptomatic and symptomatic infections of health care workers during the spring and summer of 2020 and to kinetically measure antibody levels after infection. Since SARS-CoV-2 continued circulating after the first wave in Philadelphia, we completed additional analyses to determine whether antibody levels in serum samples collected in the summer of 2020 were associated with protection from subsequent SARS-CoV-2 infections. Consistent with our recent study (3), we found that preinfection SARS-CoV-2 S-FL and N antibody levels were not associated with protection from SARS-CoV-2 infection during the fall and winter in individuals who were not infected with SARS-CoV-2 during the first viral wave. This is consistent with the observation that most prepandemic cross-reactive SARS-CoV-2 antibodies are nonneutralizing (3). Similarly, we found that preinfection OC43, HKU1, 229E, and NL63 S-FL antibody titers were not associated with protection from SARS-CoV-2 infection.

Somewhat paradoxically, we found significant negative correlations between preinfection OC43 and HKU1 antibody titers and COVID-19 symptom duration, but we did not find correlations between preinfection SARS-CoV-2 antibodies and COVID-19 symptom duration among individuals infected with SARS-CoV-2 for the first time during the second viral wave. Individuals with higher OC43 and HKU1 (both βCoVs) antibody titers experienced a shorter duration of symptoms following SARS-CoV-2 infection. Both αCoVs and βCoVs circulated during the 2019–2020 winter months in the US before the COVID-19 pandemic (21), yet the apparent cross-protection in our study appears to be specific to βCoV immunity because we found that antibody titers against the NL63 and 229E αCoVs were not associated with reducing COVID-19 symptom duration. It may seem contradictory that OC43 and HKU1 antibody levels but not SARS-CoV-2 antibody levels are associated with reduced symptom duration in individuals who are infected with SARS-CoV-2 for the first time. However, the cross-protection afforded by common βCoVs is likely not mediated by rare antibodies that cross-react to SARS-CoV-2 proteins. Instead, this protection might be mediated by cellular immune responses, which can target epitopes that are conserved among common βCoVs and SARS-CoV-2 (22). Individuals who were more recently infected with common βCoVs have higher levels of antibodies against these viruses (6), and therefore, elevated levels of antibodies against OC43 and HKU1 may serve as an immunological stamp that dates how recently an individual was exposed to common βCoVs. Additional studies need to be completed to determine the temporal relationship between recent βCoV infections and reduced symptom duration following SARS-CoV-2 infections. It is possible that T cells stimulated from recent βCoV infections (23) are involved with clearing virus and reducing symptom duration following SARS-CoV-2 infections. It is also possible that recent βCoV infections stimulate rare B cells that are quickly recalled following SARS-CoV-2 exposures or that mucosal antibodies elicited by prior CCV infections are involved in protection.

Our study has some limitations. Our health care worker cohort was not broadly representative of the human population, and it was composed of mostly young individuals. There was also a relatively small sample size of SARS-CoV-2–infected individuals in our cohort. Most infected individuals in our cohort had mild illness that was identified from an online survey, so we were unable to characterize antibody responses associated with COVID-19 hospitalizations and deaths. We only measured serum IgG antibodies in the correlate of protection part of our study, and further studies should be completed to evaluate different antibody isotypes and mucosal antibody responses. Finally, we only measured antibodies against CCV S-FL proteins, and future studies should evaluate if preinfection antibodies against other CCV proteins correlate with disease outcome following SARS-CoV-2 infection.

Moving forward, it is possible that antibody titers against OC43 and HKU1 might be useful for predicting relative infection risk among individuals who have not yet encountered SARS-CoV-2. This type of information might be important for prioritizing vaccinations while the vaccine supply remains limited. One ironic implication of our study is that individuals who have efficiently socially distanced over the past 18 months are potentially at higher risk of more severe SARS-CoV-2 symptoms, as it is unlikely that these individuals have been recently infected with common βCoVs during social isolation. Additional studies will be required to fully understand the complex relationship between CCV immunity and SARS-CoV-2 susceptibility and temporal relationships between viral infections and cross-protection.

## Methods

### Study population and data collection.

Health care workers at 3 hospitals in the University of Pennsylvania Health System (Hospital of the University of Pennsylvania, Penn Presbyterian Medical Center, and Pennsylvania Hospital) were recruited between April 13, 2020, and May 20, 2020. Only health care workers with direct contact with patients or who worked on units where patients with COVID-19 received care were included in this study. We excluded anyone who was previously diagnosed with a SARS-CoV-2 infection. Characteristics of the 2043 health care workers in our study are reported in Supplemental Table 1. We collected serum samples from each participant and quantified SARS-CoV-2 S-RBD antibodies by ELISAs within 36 to 48 hours after sample collection. We collected NP swabs from all health care workers who possessed SARS-CoV-2 S-RBD IgG and/or IgM antibodies, and we completed SARS-CoV-2 PCR testing on these samples to identify active or recent infections. Health care workers who were seronegative at baseline visit were invited for follow-up visits every 2 weeks until July 2, 2020, to identify active SARS-CoV-2 infections throughout the study period. In addition, we received serum samples from 8 health care workers who were seronegative at baseline visit and had a positive NP PCR test outside of our study. Seropositive health care workers were enrolled in a follow-up study to collect additional blood samples up to 236 days after seroconversion.

Participants filled out an online survey at time of enrollment to collect participant characteristics, including COVID-19 symptom information. A second online survey was sent to all participants in January 2021 to collect information on new SARS-CoV-2 infections that occurred after the last blood draw. Based in this information, additional SARS-CoV-2 and CCV ELISAs were completed in a subset of participants to study preinfection antibodies.

### ELISA.

ELISAs measuring antibodies against SARS-CoV-2 and against OC43, HKU1, 229E, and NL63 were completed as previously described (3, 13). SARS-CoV-2 nucleocapsid (N) protein and OC43, HKU1, 229E, and NL63 S-FL proteins were purchased from Sino Biological. Plasmids encoding SARS-CoV-2 S-FL and S-RBD were provided by Florian Krammer (Mount Sinai, New York, New York, USA). SARS-CoV-2 S-FL and S-RBD were produced in 293F cells (Thermo Fisher, R79007) and purified using Ni-NTA. Each well in an ELISA plate (Immulon 4 HBX, Thermo Scientific) was coated with 50 μL PBS or recombinant protein (2 μg/mL SARS-CoV-2 antigen or 1.5 μg/mL CCV antigen), and plates were incubated overnight at 4°C. Wells coated with only PBS were used to measure background signal for each sample. The next day, plates were washed with PBS containing 0.1% Tween-20 (PBS-T) and incubated for 1 hour with PBS-T supplemented with 3% nonfat milk powder. Heat-inactivated serum samples were diluted in PBS-T supplemented with 1% nonfat milk powder (dilution buffer). ELISA plates were washed with PBS-T, and 50 μL serum dilution was added to each well. After 2 hours of incubation, plates were washed with PBS-T and 50 μL 1:5000 diluted goat anti-human IgG-HRP (Jackson ImmunoResearch Laboratories, 109-036-098) or 1:1000 diluted goat anti-human IgM-HRP (SouthernBiotech, 2020-05) was added to each well. Plates were incubated for 1 hour and washed with PBS-T before 50 μL SureBlue TMB Substrate (KPL) was added to each well. After 5 minutes, the reaction was stopped by adding 25 μL of 250 mM hydrochloric acid. Plates were read at an optical density of 450 nm using the SpectraMax 190 microplate reader (Molecular Devices). Monoclonal antibody CR3022 (for SARS-CoV-2 S-FL and S-RBD ELISA) or an in-house created serum pool (for SARS-CoV-2 N ELISA and CCV ELISAs) was included on each plate to convert optical density values into relative antibody concentrations. Plasmids to express the CR3022 monoclonal antibody were provided by Ian Wilson (Scripps Research, La Jolla, California, USA).

### Statistics.

Health care workers with serum IgG and/or IgM concentrations above 0.48 arbitrary units/mL in SARS-CoV-2 S-RBD ELISAs were considered seropositive. This cutoff resulted in a prepandemic cross-reactive rate of 0.6% for IgG and 0.5% for IgM, as was described previously (13). SARS-CoV-2 S-RBD antibody concentrations below this cutoff at 0.48 arbitrary units/mL were assigned a value of 0.40 arbitrary units/mL. All other antibodies below the limit of detection (0.20 arbitrary units/mL) were assigned a value of 0.10 arbitrary units/mL. Antibody concentrations were log_2_ transformed for analysis, and geometric mean concentrations with 95% confidence intervals were reported unless stated otherwise. Standard descriptive analyses were used as appropriate, including the χ^2^ test, paired and unpaired 2-tailed *t* tests, Mann-Whitney test, and 1-way ANOVA with Bonferroni correction to adjust for multiple comparisons. Preexisting antibody titers were fitted to symptom durations in days in separate linear models via logistic regression with a logit link function, adjusting for age and sex. Statistical significance was set at *P* < 0.05. Prism version 9 (GraphPad) and R version 3.5.3 were used for analyses.

### Study approval.

This study was approved by the Institutional Review Board of the University of Pennsylvania under protocol 842847. Informed consent was collected from all participants before baseline visits.

## Author contributions

SG, IF, DJR, EJW, and SEH conceived and designed the study. DD, DF, AB, JW, JM, SD, and The UPenn COVID Processing Unit obtained and processed samples. SG, MEW, MJB, CPA, ECG, EMA, and CMM performed serological assays. SG and SRC analyzed data and provided statistical analyses. SG and SEH wrote manuscript with input from all authors. SEH supervised the study. IF, EJW, and SEH acquired funding.

## Supplementary Material

Supplemental data

## Version 1 07/08/2021

In-Press Preview

## Version 2 08/23/2021

Electronic publication
