# Supplementary material for: Health care worker seromonitoring reveals complex relationships between common coronavirus antibodies and COVID-19 symptom duration
Source: JCI Insight. 2021 Aug 23;6(16):e150449. doi: 10.1172/jci.insight.150449 (PMC8410018; doi:10.1172/jci.insight.150449)
Supplement: Supplemental data [file jciinsight-6-150449-s130.pdf]

Supplemental Acknowledgments: We thank members of the UPenn COVID Processing Unit: Sharon Adamski, Zahidul Alam, Mary M. Addison, Katelyn T. Byrne, Aditi Chandra, Kurt D'Andrea, Hélène C. Descamps, Nicholas Han, Yaroslav Kaminskiy, Shane C. Kammerman, Justin Kim, Allison R. Greenplate, Jacob T. Hamilton, Nune Markosyan, Julia Han Noll, Dalia K. Omran, Ajinkya Pattekar, Eric Perkey, Elizabeth M. Prager, Dana Pueschl, Austin Rennels, Jennifer B. Shah, Jake S. Shilan, Nils Wilhausen, and Ashley N. Vanderbeck.

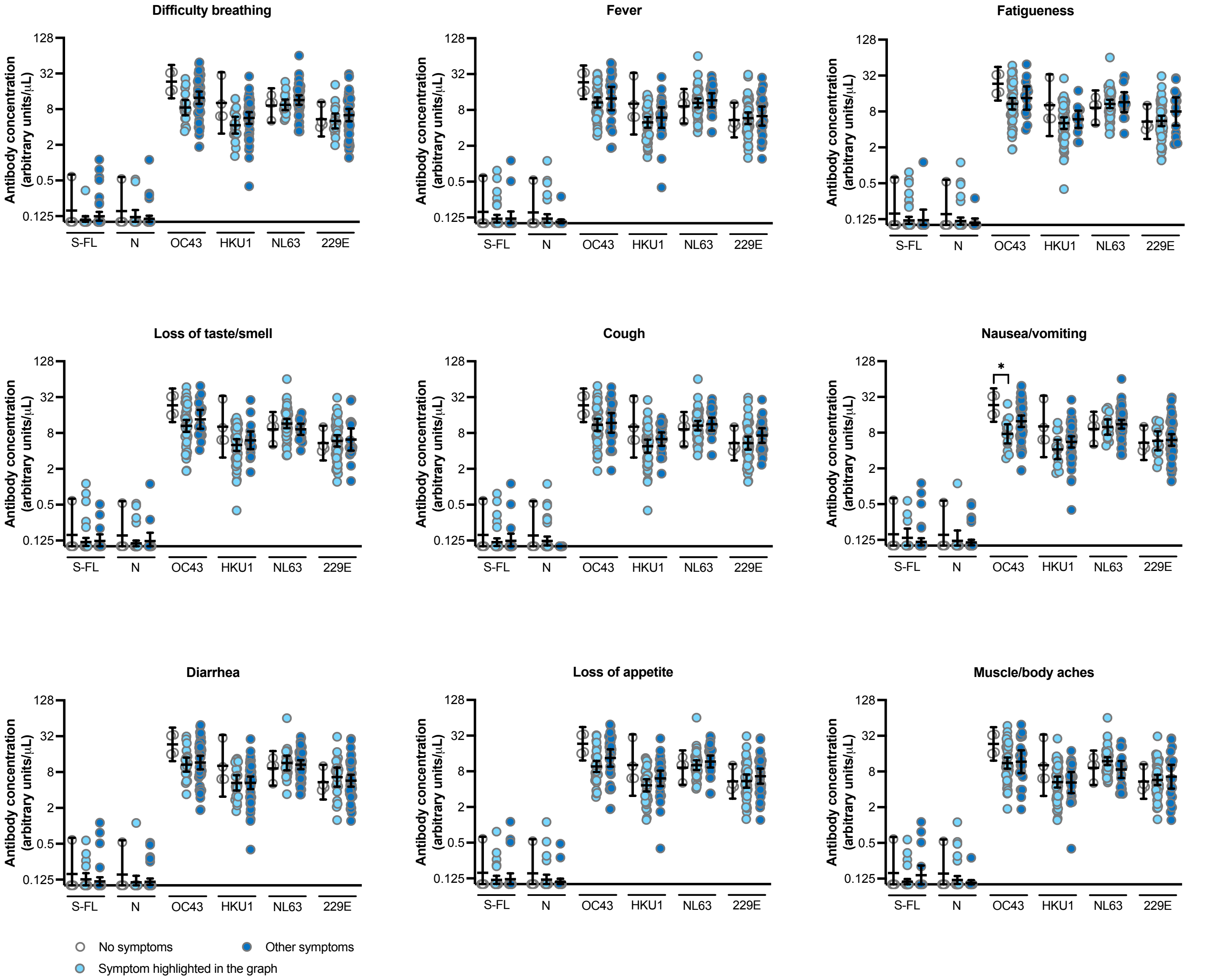

Difficulty breathing
Fever
Fatigueness
Loss of taste/smell
Cough
Nausea/vomiting
Diarrhea
Loss of appetite
Muscle/body aches
Antibody concentration (arbitrary units/μL)
128
32
8
2
0.5
0.125
S-FL
N
OC43
HKU1
NL63
229E
*
No symptoms
Other symptoms
Symptom highlighted in the graph

Supplemental Figure. Correlation between pre-existing antibody concentrations and reported COVID-19-related symptoms in symptomatic (n=64) and asymptomatic (n=4) health care workers with a PCR-confirmed SARS-CoV-2 infection. Significant p-values (<0.05) are indicated above the graph (one-way ANOVA using log_2-transformed antibody concentrations). Horizontal lines show the geometric mean concentrations and 95% confidence intervals. * p<0.05.

Supplemental Table 1. Participant characteristics.

	Total (N=2043)	Seropositive (N=55)	Seronegative (N=1988)	P-value
Age (in years), median (IQR)	36 (30-46)*	34 (29-44)	36 (30-46)*	0.293
Sex, n (%)				0.719
Male	502 (24.6)	17 (30.9)	485 (24.4)	
Female	1536 (75.2)	38 (69.1)	1498 (75.4)	
Other	2 (0.1)	0	2 (0.1)	
Unknown	3 (0.1)	0	3 (0.2)	
Race, n (%)				0.291
Asian	172 (8.4)	2 (3.6)	170 (8.6)	
Black	117 (5.7)	5 (9.1)	112 (5.6)	
White	1694 (82.9)	45 (81.8)	1649 (82.9)	
Other/multiracial	33 (1.6)	1 (1.8)	32 (1.6)	
Unknown	27 (1.3)	2 (3.6)	25 (1.3)	
Ethnicity, n (%)				0.293
Hispanic	71 (3.5)	4 (7.3)	67 (3.4)	
Non-Hispanic	1971 (96.5)	51 (92.7)	1920 (96.6)	
Unknown	1 (<0.1)	0	1 (0.1)	

* Age was not reported for 1 participant.

Supplemental Table 2. Characteristics of 64 participants who had no detectable S-RBD antibodies during the spring and summer of 2020 and reported via the online survey that they had a symptomatic lab-confirmed SARS-CoV-2 infection after the last blood draw.

	**Total (N=64)**
Symptoms, n (%)	
Cough	42 (65.6)
Fever or chills	42 (65.6)
Nausea/vomiting	13 (20.3)
Diarrhea	20 (31.3)
Unusual fatigue	51 (79.7)
Loss of taste or smell	47 (73.4)
Difficulty breathing	17 (26.6)
Loss of appetite	34 (53.1)
Muscle/body aches	45 (70.3)
Other	23 (35.9)
Estimated date of SARS-CoV-2 infection, n (%)	
July 2020	6 (9.4)
August 2020	1 (1.6)
September 2020	0
October 2020	9 (14.1)
November 2020	14 (21.9)
December 2020	30 (46.9)
January 2021	4 (6.3)
Resolution of symptoms, n (%)	
Within 7 days	13 (20.3)
Within 1 month	32 (50.0)
More than 1 month	13 (20.3)
Unknown	6 (9.4)
Hospitalization because of COVID-19, n (%)	2 (3.1)

Supplemental Table 3. Effects of log_2 pre-existing antibody titers, age and sex on symptom duration (days) via multivariate regression.

	**Estimate**	**SE**	**P-value**
S-FL	-2.731	2.685	0.313
Age	3.204	2.697	0.240
Sex (male)	5.675	7.964	0.479
N	1.895	2.698	0.485
Age	3.606	2.712	0.189
Sex (male)	6.299	7.997	0.434
OC43	-8.166	2.739	0.004
Age	0.170	2.752	0.951
Sex (male)	4.226	7.498	0.575
HKU1	-5.867	2.630	0.030
Age	2.392	2.644	0.369
Sex (male)	5.559	7.704	0.474
NL63	-2.354	2.683	0.384
Age	3.548	2.697	0.194
Sex (male)	6.218	7.973	0.439
229E	-3.054	2.846	0.288
Age	2.840	2.742	0.305
Sex (male)	8.483	8.255	0.308
